# Adhesive Hydrogels as Fixation and Regeneration Platforms in Cartilage Surgery: Rethinking Scaffold-Tissue Integration from a Clinical Perspective

**DOI:** 10.3390/ijms27104600

**Published:** 2026-05-20

**Authors:** Hyejin Jo, Seunghun S. Lee

**Affiliations:** Department of Biomedical Engineering, Dongguk University, Seoul 04620, Republic of Korea

**Keywords:** adhesive hydrogel, bioadhesive, cartilage repair, tissue integration, scaffold fixation, injectable hydrogel, chondrogenesis

## Abstract

Articular cartilage defects affect millions of patients annually and pose one of the most persistent challenges in orthopedic surgery, owing to the tissue’s inherent avascular and alymphatic nature. Current surgical approaches, microfracture, autologous chondrocyte implantation (ACI/MACI), and osteochondral grafting, share a common failure mode: inadequate adhesion between repair constructs and surrounding native cartilage, contributing to deterioration rates of 15–75% at five-year follow-up across all techniques. This review repositions adhesion not as a supplementary material property but as the central determinant of clinical success in cartilage repair. We systematically evaluate the biomechanical demands imposed by the joint environment and define clinically relevant adhesion thresholds. Adhesive hydrogel strategies are categorized by surgical context: microfracture augmentation, ACI/MACI enhancement, osteochondral graft integration, and standalone repair platforms. Material platforms are analyzed across catechol/dopamine systems, NHS ester chemistry, photocrosslinkable hydrogels, supramolecular approaches, and multi-mechanism hybrids. Injectable formulations for arthroscopic delivery are critically examined alongside key translational barriers, including fatigue durability, biocompatibility–adhesion trade-offs, sterilization compatibility, batch variability, and regulatory classification ambiguity. Future directions encompass 4D bioprinting, AI-guided formulation optimization, and stimuli-responsive reversible adhesion systems. Adhesive hydrogels represent the missing link that current cartilage repair paradigms require.

## 1. Introduction

Articular cartilage injury remains one of the most challenging clinical problems in orthopedic surgery, with cartilage lesions of the knee affecting approximately 900,000 patients annually in the United States and accounting for more than 200,000 surgical procedures each year [1,2]. These injuries span a broad spectrum of etiologies, including acute traumatic injuries from sports or accidents, degenerative joint disease, osteochondral defects, and post-traumatic osteoarthritis [1]. The clinical significance of cartilage defects extends well beyond immediate morbidity; untreated lesions progress to osteoarthritis in up to 50% of cases within 10 years [3], imposing a substantial socioeconomic burden estimated at over USD 136 billion annually in the United States alone [4]. Despite advances in surgical technique and biomaterial science, the fundamental challenge remains unchanged: achieving durable integration between repair tissue and surrounding native cartilage while restoring load-bearing functionality [3,5].

The biological constraints of cartilage healing represent the root cause of this persistent clinical failure. Unlike vascularized tissues, articular cartilage is avascular, aneural, and alymphatic—characteristics that confer its unique load-bearing properties but simultaneously preclude the inflammatory response and healing cascade observed in other tissues [3,5]. Adult articular cartilage exhibits extremely low cellularity (approximately 100–200 cells/mm^3^) and minimal metabolic activity relative to tissue volume [3]. Chondrocytes exist in a relatively static state, with limited capacity for proliferation and matrix remodeling. Once cartilage is damaged, the absence of vascularization prevents the recruitment of progenitor cells and inflammatory cells necessary to initiate repair [6]. Consequently, untreated full-thickness cartilage defects do not spontaneously heal; instead, they progressively enlarge through mechanical wear and biochemical degradation of residual extracellular matrix [3,6].

Current surgical approaches attempt to circumvent cartilage’s limited healing capacity through diverse mechanistic strategies, yet all share a common failure point: inadequate fixation and integration of repair tissue with surrounding native cartilage [6]. Microfracture, the simplest and most economical approach, relies on stimulating marrow bleeding from subchondral bone to create a fibrin clot scaffold [7]. While initial outcomes may be promising, the fibrin clot is rapidly degraded by synovial fluid enzymes and mechanical washout, leading to fibrocartilage formation rather than true hyaline cartilage repair [6,8]. Autologous chondrocyte implantation (ACI) and its matrix-assisted variant (MACI) demonstrate superior cartilage quality but suffer from periosteal flap delamination, incomplete lateral integration, and cell leakage during the immediate postoperative period [9,10,11]. Osteochondral grafting provides structural support but leaves unfilled gaps between graft and host tissue, compromising mechanical integrity and promoting degenerative changes [12,13]. The common denominator across all approaches is inadequate adhesion—both to maintain structural integrity and to establish biomechanical continuity with native cartilage [6,14].

Adhesive technologies have transformed surgical practice in otolaryngology, ophthalmology, dermatology, and maxillofacial surgery [15]. Fibrin sealants have become standard adjuncts in hemostasis and wound closure, while cyanoacrylates provide rapid bonding in microsurgery. The dental field has extensively developed adhesive systems for restorative dentistry, achieving bond strengths exceeding 20–30 MPa [15]. However, cartilage repair surgery has not benefited from these adhesion paradigms, relying instead on mechanical fixation devices (sutures, anchors) or scaffold materials that passively fill space without actively bonding to host tissue [6,15]. This represents a significant gap in surgical technology: while adhesion is recognized as essential in other tissues, it remains largely unaddressed in cartilage surgery [6].

Hydrogels-three-dimensional polymer networks with water content exceeding 90%-have emerged as the dominant scaffold platform in tissue engineering due to their biocompatibility, tunable mechanical properties, and capacity for biomolecule incorporation [16,17]. Recent advances have transformed hydrogels from inert passive scaffolds to active biological platforms through the incorporation of adhesive functional groups [15,16]. Adhesive hydrogels combine the biological advantages of hydrogel materials with the mechanical benefit of wet tissue adhesion, addressing the integration challenge that has plagued cartilage surgery. These materials employ diverse adhesion mechanisms, ranging from mussel-inspired catechol chemistry to NHS ester-mediated covalent bonding, enabling robust fixation under physiological conditions. The field has advanced rapidly; over 200 peer-reviewed publications on adhesive hydrogels have appeared since 2015, with accelerating development of cartilage-specific formulations [16,17].

This review examines adhesive hydrogels from an explicitly clinical perspective, asking how these materials address the real, quantifiable failures of current cartilage repair approaches. Rather than surveying hydrogel adhesion broadly, we specifically focus on platforms designed for or adaptable to cartilage defect repair in clinical settings. We analyze the biomechanical requirements imposed by the joint environment, categorize adhesive strategies by surgical application (augmenting microfracture, enabling ACI without periosteal flap, filling osteochondral graft gaps, or serving as standalone repair matrices), and critically examine material platforms and delivery mechanisms. We then evaluate the substantial translational barriers, adhesion durability under cyclic loading, regulatory ambiguity, sterilization compatibility, and animal model limitations that currently prevent clinical translation. Throughout, we emphasize that adhesion is not a supplementary feature but rather the missing link that determines clinical success or failure in cartilage tissue engineering.

## 2. Clinical Landscape of Cartilage Repair and Integration Challenges

### 2.1. Microfracture: Marrow Stimulation and Clot Instability

Microfracture represents one of the most commonly performed first-line cartilage repair procedures worldwide [18]. The technique involves arthroscopic penetration of the subchondral bone plate with specialized awls, spaced 3–4 mm apart, to a depth of 2–4 mm [7]. This controlled fracture of subchondral bone triggers bleeding, establishing a fibrin clot scaffold that gradually transitions to fibrocartilage and eventually hyaline cartilage over 12–24 months [7]. Initial clinical results are often encouraging; multiple studies report pain reduction and functional improvement at 2-year follow-up in 70–85% of patients [7]. However, long-term outcomes reveal substantial deterioration: hyaline cartilage formation occurs in only 25–50% of cases at 5-year follow-up, while 50–75% develop predominantly fibrocartilage with inferior mechanical properties [8,18].

The adhesion failure in microfracture occurs at two critical interfaces. First, the fibrin clot itself is inherently unstable within the joint environment [8,19]. Synovial fluid contains matrix metalloproteinases (MMPs), tissue plasminogen activator (tPA), and other proteolytic enzymes that rapidly degrade fibrin. Shear stresses generated during joint motion, ranging from 0.5 to 5 MPa, mechanically displace the clot in the absence of tissue adhesion [8,20]. The clot washout phenomenon is well-documented in experimental studies; fibrin scaffolds are substantially degraded within 48 h of exposure to synovial fluid enzymes, with fibrin clot scaffolds showing complete degradation within 12–24 h under MMP-1 conditions [20]. Second, even if the clot remains present, the interface between repair tissue and native cartilage demonstrates incomplete integration [6,8]. The repair tissue forms a fibrocartilage layer with substantially different mechanical properties than native hyaline cartilage, creating a stiff interface discontinuity that concentrates stress and promotes delamination.

### 2.2. Autologous Chondrocyte Implantation and Periosteal Flap Delamination

ACI, pioneered by Brittberg et al. in the 1990s, delivers expanded autologous chondrocytes suspended in solution into a cartilage defect, with the cellular construct initially secured by a periosteal flap sutured to the defect margins [10,11]. Third-generation MACI replaces the periosteal flap with a more refined collagen membrane scaffold, improving cell retention and reducing donor-site morbidity. This approach demonstrates superior hyaline cartilage formation compared to microfracture; histological studies show that approximately 90% of treated defects exhibit hyaline or hyaline-like cartilage characteristics at 12–24 months [9].

The adhesion challenge in ACI/MACI exists at three distinct levels. First, the periosteal flap itself is prone to delamination from the cartilage surface, with reported rates of 5–26% depending on defect location and surgeon experience [9,14]. This delamination is not simply a technical failure but reflects fundamentally inadequate biological adhesion between periosteal tissue and hyaline cartilage, tissues with distinct biochemical compositions and divergent mechanical properties [14]. Second, cell leakage remains problematic in the early postoperative period; chondrocytes can escape from the defect into the surrounding synovial fluid, reducing the viable cell population available for repair [8]. While third-generation MACI with matrix scaffolds provides improved cell retention, the lateral integration between repair tissue and surrounding native cartilage remains incomplete. Scanning electron microscopy and histological analyses consistently reveal a persistent interface gap between regenerated and native cartilage at 12 months, creating a zone of mechanical discontinuity that concentrates stress and limits long-term durability [14].

### 2.3. Osteochondral Autograft Transfer System: Gap-Filling and Dead Space

Osteochondral autograft transfer system (OATS) involves harvesting intact osteochondral plugs—cylindrical grafts containing both cartilage and underlying subchondral bone—from non-weight-bearing regions of the knee and implanting them into the defect site [12,21]. The technique offers structural support and vascular access through the bone component, theoretically enabling superior integration compared to purely cartilaginous repair approaches. Short-term clinical outcomes (2–3 years) are favorable, with return to sport reported in 60–75% of patients. However, medium-term failure rates are substantial: radiographic evidence of graft loosening, subsidence, or degenerative changes occurs in 20–40% of cases by 5-year follow-up.

The adhesion failure in OATS is geometrically intrinsic to the procedure. Even with precise defect reaming and plug insertion, gaps inevitably remain between graft edges and host cartilage, typically measuring 100–500 μm depending on reaming precision [12]. These unfilled spaces are rapidly populated by synovial fluid and inflammatory cells, preventing biological adhesion between graft and host tissue. Moreover, the graft experiences mechanical micromotion at the interface, generating shear stresses that further impair integration. The dead space at the graft-host interface represents a zone of mechanical discontinuity that concentrates stress, contributing to progressive degradation of the repair construct.

### 2.4. Fresh Osteochondral Allograft Transplantation: Immunologic and Integration Barriers

**Figure 1:**
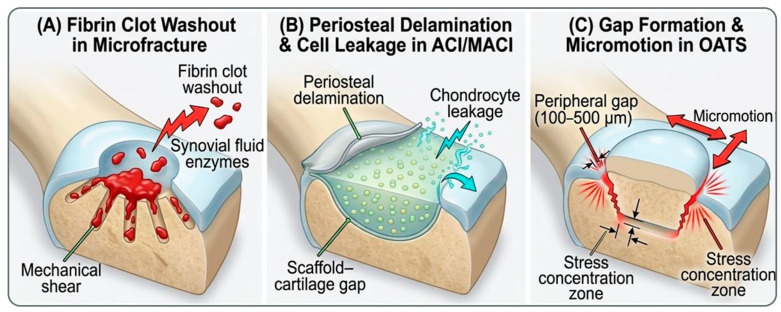
Adhesion failure mechanisms in current cartilage repair approaches. (**A**) Fibrin clot washout by synovial fluid enzymes and mechanical shear in microfracture. (**B**) Periosteal flap delamination and chondrocyte leakage in ACI/MACI. (**C**) Peripheral gap formation (100–500 µm) and mechanical micromotion at the graft–host interface in OATS.

Fresh osteochondral allograft transplantation utilizes size-matched cadaveric osteochondral grafts to address large (>2 cm^2^) or complex defects that exceed the reconstructive capacity of autologous techniques [13]. Unlike autografts, allografts provide structural bone support with viable hyaline cartilage containing intact chondrocytes within a preserved extracellular matrix. However, this approach introduces additional integration challenges beyond those observed with autologous grafts [13,14]. Gap formation between allograft and host tissue mirrors the geometric mismatch inherent to OATS procedures, with peripheral dead space compromising lateral integration [12,13]. Additionally, immunologic concerns, including chondrocyte viability loss during storage and potential host immune responses to allogeneic tissue, compound the integration challenge. Fresh allografts must be implanted within 28 days of procurement to maintain chondrocyte viability; mandatory serologic and microbiologic testing periods typically consume 14 of those available days, leaving a narrow window for size-matching and scheduling [13,22]. Even within this constrained timeline, cold storage at 4 °C results in progressive chondrocyte death with viability declining by approximately half within the initial 14-day clearance period, further compromising graft quality independent of immunologic factors (Figure 1) [23,24].

### 2.5. Summary: Integration Failure as the Common Denominator

Across all established cartilage repair approaches, inadequate adhesion at critical interfaces emerges as the primary determinant of clinical failure. Whether examining fibrin clot stability in microfracture, periosteal flap or membrane adhesion in ACI or graft-host integration in osteochondral surgery, the pattern is consistent: materials and tissues that do not actively bond to surrounding structures progressively delaminate and fail (Table 1). The clinical consequence is predictable; a substantial proportion of cartilage repair procedures demonstrate significant deterioration by 5-year follow-up, regardless of initial technique. This uniform failure pattern across diverse surgical approaches points to a fundamental technical gap, the absence of adequate adhesion, rather than a deficiency inherent to any single surgical method.

Current surgical practice addresses this limitation primarily through mechanical fixation devices such as sutures, anchors, and screws, rather than through biological adhesion. Mechanical fixation increases operative time, requires additional incisions or instrumentation, and carries risks of tissue damage from anchoring devices. Even with optimized mechanical fixation, the underlying adhesion problem remains unaddressed. The missing paradigm in cartilage surgery, one already well established in other surgical specialties, is active biological adhesion that bonds repair materials directly to the cartilage surface, stabilizing the repair construct against synovial fluid washout and mechanical loading while simultaneously promoting biological integration.

## 3. Biomechanical Requirements for Cartilage Adhesion

### 3.1. Joint Loading Environment and Physiological Stresses

The joint environment imposes formidable mechanical demands on adhesive systems. The knee joint, the most commonly repaired site, experiences highly variable loading patterns throughout the gait cycle and during diverse activities [25]. During normal walking at a self-selected pace, compressive stress at the patellofemoral joint peaks at approximately 1–1.5 MPa while tibiofemoral compartment stress reaches 2–3 MPa. More intense activities dramatically escalate these loads: stair climbing increases compressive stress to 3–6 MPa, and jumping or landing maneuvers generate peak stresses exceeding 10–18 MPa in the tibiofemoral compartment. These are not static loads but dynamic, cyclic stresses repeated thousands of times daily; the cumulative mechanical burden represents one of the most demanding loading environments encountered in the human body [25].

Beyond compression, shear stresses represent the dominant mechanical challenge for adhesion in articulating joints. During joint motion, the articular surfaces slide relative to one another, generating tangential forces at the cartilage surface. In vivo measurements indicate shear stress magnitudes of 0.5–2 MPa during walking and up to 5 MPa during pivoting or cutting maneuvers [25]. These shear stresses directly oppose adhesion, generating tensile stress at the adhesive interface that can exceed the adhesion strength of most materials. The synovial fluid environment further complicates adhesion; the aqueous milieu creates a hydration layer between tissue surfaces that must be overcome for effective wet adhesion [26,27].

**Figure 2:**
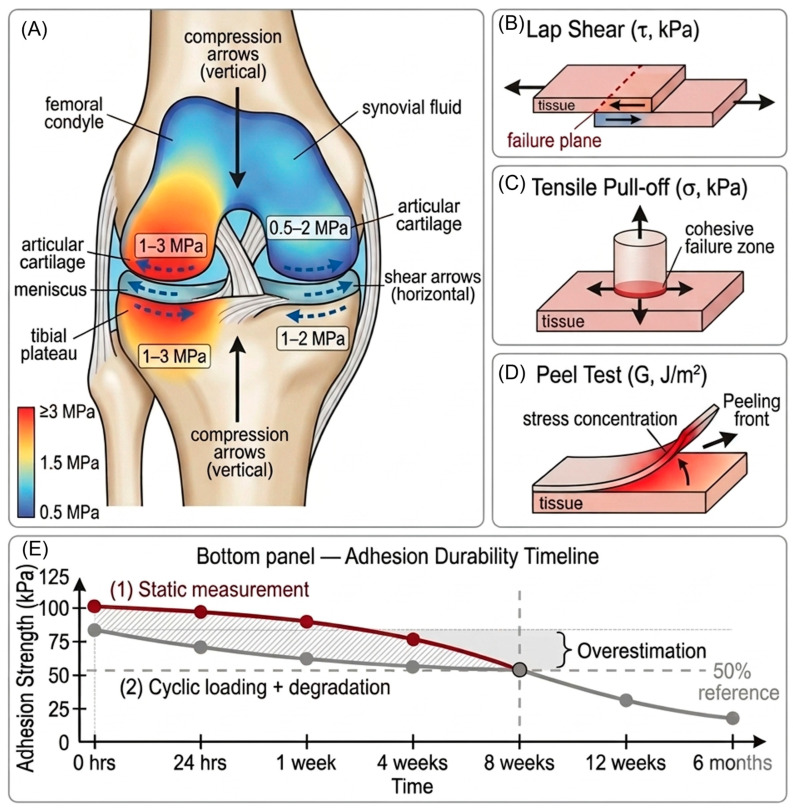
Biomechanical environment and adhesion testing paradigms. (**A**) Compressive (1–3 MPa) and shear (0.5–2 MPa) stress distribution during weight-bearing. (**B**) Lap shear test. (**C**) Tensile pull-off test. (**D**) Peel test. (**E**) Adhesion durability timeline: static single time-point measurement (curve 1) versus progressive strength loss under cyclic loading and degradation (curve 2).

Synovial fluid itself contains components actively antagonistic to adhesion. MMPs, particularly MMP-2 and MMP-9, are continuously present in synovial fluid and increase substantially with joint inflammation, degrading collagen and proteoglycans that constitute primary components of most biological adhesives [28]. Additionally, synovial fluid contains tPA and other fibrinolytic enzymes that rapidly dissolve fibrin-based adhesives [20]. The pH of synovial fluid (7.2–7.4) and ionic strength mimic plasma but differ sufficiently to alter the behavior of many polyelectrolyte systems. Synovial fluid also contains hyaluronic acid (HA) at concentrations of 2–4 mg/mL and various proteoglycans that adsorb to material surfaces, potentially preventing the intimate tissue–material contact necessary for adhesion (Figure 2) [26].

### 3.2. Adhesion Strength Metrics and Clinical Thresholds

Defining clinically adequate adhesion strength requires synthesis of biomechanical data and standards from analogous surgical fields. Multiple standardized adhesion testing methods exist, each measuring different aspects of bond strength [15]. Lap shear tests measure the force required to rupture adhesive bonds between two overlapping surfaces under pure shear stress, typically reported in kilopascals (kPa). Tensile pull-off tests (butt joint tests) measure adhesive strength under perpendicular tensile stress using cylindrical geometries. Peel tests measure the force per unit width required to separate bonded surfaces at controlled angles, providing particularly relevant data for thin adhesive interfaces. These adhesion test methods and their clinical relevance are summarized in Table 2.

Translating these measurements to clinical requirements demands consideration of several factors. Adhesion testing typically measures acute bond strength immediately after adhesive maturation, yet clinical requirements extend over years during which adhesive systems degrade, and fatigue cycling occurs [28,29]. A conservative approach suggests that sufficient adhesion strength must exceed the maximum physiological stresses imposed by the joint environment. For microfracture augmentation or ACI-supporting platforms, which must resist predominantly shear stress from joint motion, adhesion strength exceeding 50–100 kPa is likely necessary [27]. For osteochondral graft fixation, where adhesion must resist both compression and delamination during weight-bearing, higher thresholds of 100–200 kPa may be required.

In vivo instrumented-prosthesis studies of the human knee report cartilage-surface compressive stresses of 1–3 MPa during normal walking and peak shear stresses of 0.3–1.0 MPa during gait, rising to 1.5–2.0 MPa during stair descent and pivot maneuvers [23,30]. Applying a minimum 2× safety factor consistent with ASTM F2255 conventions for tissue-bonding adhesives, the threshold values stated above should therefore be interpreted as follows. (i) Microfracture-augmentation systems, which protect a maturing fibrin/marrow clot during a reduced-weight-bearing rehabilitation window, require a preliminary engineering target of 50–100 kPa shear strength. (ii) ACI/MACI-supporting platforms, which must resist predominantly tangential shear during early weight-bearing, require a preliminary engineering target of 100–200 kPa. (iii) OATS gap-filling systems, which must bridge graft-host interfaces under 0.5–1.0 MPa worst-case interfacial shear, require a preliminary target of 200–500 kPa after the 2× safety factor is applied. We emphasize that these are preliminary engineering targets derived from joint-mechanics extrapolation, not clinically validated thresholds; future studies should report adhesion strength as a fraction of the local in vivo stress envelope rather than as an absolute kPa figure.

Standards from adjacent surgical fields provide useful benchmarks. Dental adhesive systems, which also bond to mineralized tissue under aqueous conditions, routinely achieve bond strengths of 20–30 MPa, although these operate under very different conditions than cartilage repair (non-load-bearing, minimal enzymatic environment, smaller defects) [15]. Skin adhesives used in wound closure typically achieve adhesion strengths of 5–10 kPa but are not designed for load-bearing applications [15]. Fibrin sealants, the primary adhesive used in orthopedic surgery, demonstrate tensile strengths below 8 kPa and shear strengths below 30 kPa in ex vivo cartilage models, values consistently insufficient for load-bearing fixation [15,29]. Failure mode analysis adds important nuance to adhesion strength interpretation. Adhesive failure, where the bond ruptures at the material–tissue interface, indicates insufficient adhesion strength and is always undesirable [15]. Cohesive failure, where the adhesive material itself ruptures while the interface remains intact, may indicate adequate interfacial bonding with the limiting strength residing in the bulk material. For cartilage repair applications, mixed-mode failure combining both mechanisms is considered optimal, indicating that interface bonding strength exceeds bulk material strength.

### 3.3. Additional Requirements Beyond Strength

Adhesion strength measured acutely provides only partial information about clinical adequacy. Fatigue resistance, the ability to maintain adhesion under repeated cyclic loading, is equally critical yet consistently neglected in adhesive hydrogel studies [29]. Fatigue damage in adhesive joints occurs through cyclic crack initiation and propagation at the interface or within the bulk adhesive, even under stresses below the static failure threshold. Most adhesive hydrogel studies lack fatigue data; the limited studies that include fatigue testing report substantial strength reduction after 100,000 loading cycles, equivalent to approximately two weeks of physiological activity, indicating that acute adhesion strength measurements substantially overestimate long-term clinical durability [28,31].

Adhesion maintenance during material degradation represents another critical requirement absent from most adhesive hydrogel studies. All hydrogels eventually degrade, through hydrolysis, enzymatic cleavage, or cellular remodeling, over timescales ranging from weeks to months [28]. As the bulk hydrogel degrades and the material loses mass, interfacial stresses increase; the bond must remain functional throughout this degradation period. Preliminary evidence suggests that adhesion strength often declines substantially as material mass loss progresses, implying that adhesion at the 6-month timepoint, typical of cartilage repair timelines, may be substantially lower than adhesion measured immediately after implantation [31].

The synovial fluid environment continuously challenges adhesion through enzymatic attack, dilution effects, and biochemical competition. Most in vitro adhesion testing is conducted in simple buffers or phosphate-buffered saline (PBS), which inadequately replicate the enzymatic and protein-rich nature of synovial fluid. Studies examining adhesion in synovial fluid or enzyme-containing media report markedly reduced adhesion strength compared to PBS controls, a factor that is frequently underappreciated but carries major implications for predicting clinical performance [28,29].

## 4. Adhesive Hydrogel Strategies Categorized by Surgical Application

### 4.1. Adhesive Hydrogels for Microfracture Augmentation

Microfracture augmentation represents the most straightforward clinical application for adhesive hydrogels: stabilizing the fibrin clot scaffold and preventing washout by synovial fluid. BST-CarGel (Anika Therapeutics, MA, USA) is the first adhesive hydrogel system to receive regulatory approval (Health Canada, CE mark) for clinical use in cartilage repair [32]. The product consists of chitosan, a positively charged biopolymer derived from crustacean shells, suspended in glycerol phosphate solution [33]. Upon exposure to physiological pH and temperature, chitosan–glycerol phosphate (chitosan-GP) undergoes thermo-responsive gelation, forming a hydrogel that physically traps fibrin clot and localizes growth factors. More importantly, chitosan’s positive charge enables electrostatic interaction with the negatively charged cartilage surface (rich in glycosaminoglycans and proteoglycans), generating reversible adhesion.

Phase III clinical trial data support BST-CarGel’s efficacy. The landmark study by Stanish et al. compared microfracture alone versus microfracture augmented with BST-CarGel in 80 patients with cartilage defects [32]. At 12-month follow-up, arthroscopic biopsies showed significantly superior hyaline cartilage formation in the BST-CarGel group (68% of biopsies predominantly hyaline cartilage) versus control microfracture (48% predominantly hyaline cartilage). Notably, at 5-year follow-up, BST-CarGel treatment maintained significantly superior repair tissue quantity and quality compared with microfracture alone, demonstrating durable structural benefit over the mid-term [32,34]. The mechanism of BST-CarGel involves both mechanical stabilization (preventing clot washout) and biological enhancement (chitosan promotes chondrogenic differentiation of marrow-derived mesenchymal stem cells (MSCs) through TLR4 signaling) [35].

GelrinC (formerly Geneasis, BioMimetrics Inc.) employs a photocrosslinkable PEG-fibrinogen scaffold concept first described by Almany and Seliktar, in which fibrinogen domains anchor the construct to native fibrin clot while UV-initiated PEG crosslinking stabilizes the gel structure [36]. Upon mixing, fibrinogen peptides bind to the fibrin clot, and UV light triggers PEG crosslinking, covalently bonding the hydrogel to the clot while simultaneously stabilizing clot structure. Early clinical data (Phase II trials) showed efficacy comparable to BST-CarGel, with hyaline cartilage formation in approximately 65% of treated defects at 12 months [37]. However, GelrinC requires intraoperative UV light delivery, adding procedural complexity and potential phototoxicity concerns, limiting wider clinical adoption.

Emerging microfracture augmentation systems employ alternative chemistries. HA-based adhesive hydrogels conjugated with reactive groups (aldehyde or catechol) bind electrostatically and covalently to the cartilage surface while stabilizing the fibrin clot [38]. Alginate-based systems similarly provide both adhesion and clot stabilization. The key design principle unifying all successful microfracture augmentation systems is dual functionality: maintaining fibrin clot integrity while actively bonding to the cartilage surface to prevent material washout [15,28].

### 4.2. Adhesive Hydrogels for Aci/Maci Enhancement

ACI/MACI surgery has evolved toward reducing or eliminating the need for periosteal flap coverage, which introduces an additional surgical step and risks periosteal complications [10,14]. Adhesive hydrogels enable this evolution by replacing the periosteal flap with adhesive sealant or adhesive membrane [39]. Instead of suturing a periosteal flap over the defect edge, the surgeon applies a thin layer of adhesive hydrogel to cartilage margins, bonding it in place and creating a physical barrier against cell leakage.

Several approaches have been explored. Fibrin-based adhesive systems modified with RGD peptides (cell adhesion motifs) and TGF-β incorporation combine adhesion with controlled growth factor delivery to enhance chondrogenic differentiation within implanted cell populations [40]. HA-peptide adhesive systems utilizing mussel-inspired dihydroxyphenylalanine (DOPA) chemistry create robust adhesion to cartilage (which is rich in HA-binding proteins) while providing three-dimensional space for cell proliferation [39,41]. The adhesive coating approach involves applying a thin adhesive layer to periosteal or collagen membrane prior to implantation, using the adhesive to bond the membrane to the cartilage edge and prevent delamination.

Cell-laden adhesive hydrogels represent a more ambitious approach: embedding chondrocytes or MSCs directly within the adhesive hydrogel rather than using periosteal membrane [31]. This one-step approach combines cell delivery with adhesion in a single implant, potentially reducing operative complexity. However, this strategy faces significant challenges: incorporating cells requires biocompatible gelation conditions (mild pH, low temperature, absence of toxic crosslinkers), yet these same mild conditions often compromise adhesion strength compared to acellular systems. The cell-laden approach also complicates regulatory approval, as cell-containing products are classified as advanced therapy medicinal products (ATMPs) requiring significantly more complex regulatory pathways [42].

#### Cell-Laden Adhesive Hydrogels: Why Cells Matter for Tissue Integration

From a surgical standpoint, embedding cells within an adhesive matrix is not a regulatory burden to be avoided but a biological imperative for durable integration. A surgeon implanting an acellular adhesive scaffold relies entirely on host–cell migration from the marginal zone of the host cartilage, a tissue that is itself avascular, alymphatic, and chondrocyte-sparse. This biologically unreliable assumption underlies the long-term integration failure of every acellular cartilage-repair platform reviewed in Section 2. Cell-laden adhesive hydrogels directly remedy this dependence by populating the construct with chondrocytes or MSCs at the moment of implantation, ensuring that matrix synthesis begins immediately rather than awaiting an uncertain host–cell-recruitment phase.

The interface-remodeling argument is equally fundamental. Chondrocytes within an adhesive hydrogel actively secrete collagen II, aggrecan, and lubricin at the construct–host boundary, producing a biological interface—a true “marriage” of construct and tissue, in the sense Reviewer #2 used the term—that no acellular system can generate. Long-term durable integration requires this interfacial extracellular-matrix remodeling, which is in turn the substrate on which adhesive forces transition from chemical (catechol, NHS, electrostatic) to biological (collagen continuity, proteoglycan interpenetration). In our analysis, no acellular system currently described in the literature can recapitulate this transition; cell-laden adhesive hydrogels are therefore not an alternative modality but the modality most directly aligned with the biology of cartilage repair.

Two surgical workflows are emerging. The one-step intraoperative protocol delivers chondrocytes or MSCs suspended in an injectable adhesive precursor, with arthroscopic in situ gelation; this minimizes operative time, anesthesia exposure, and donor-site morbidity, and is conceptually compatible with autologous, allogeneic, or banked cell sources. The two-step protocol mirrors MACI/ACI: chondrocyte harvest, ex vivo expansion, and re-implantation in an adhesive matrix; this incurs an additional operative step but allows GMP cell-quality control. The one-step approach is more attractive for routine arthroscopic use, while the two-step approach better matches the existing regulatory infrastructure for cell therapies.

The regulatory dimension is a tractable design constraint rather than a reason to deprioritize the modality. Three viable regulatory routes exist for cell-laden adhesive constructs: (i) the FDA Biologics License Application (BLA) pathway under CBER + 21 CFR 1271, with the MACI^®^ approval (Vericel, MA, USA, 2016) as the established precedent; (ii) the EMA centralized Advanced Therapy Medicinal Product (ATMP) pathway under the Tissue-Engineered Product subcategory, with Spherox^®^ (CO.DON, Germany, 2017) as the most direct precedent; and (iii) the Japanese conditional Regenerative Medicine Act (PMD Act 2014), which allows market access with a 7-year provisional license pending efficacy confirmation, with JACC^®^ (Japan Tissue Engineering, Japan 2012) as the primary cartilage-product precedent [43]. The ChondroCelect withdrawal (2014) provides a cautionary precedent for reimbursement and manufacturing complexity but does not negate the regulatory feasibility of the modality. Cell-laden adhesive hydrogels therefore deserve equal scientific weight to acellular systems in this review, and the surgical advantages—biological interface remodeling, immediate matrix synthesis, and the possibility of one-step intraoperative delivery—argue for their continued preclinical development as the most biologically aligned modality for long-term cartilage integration.

### 4.3. Adhesive Hydrogels for Osteochondral Graft Integration

Osteochondral grafting presents unique adhesion challenges because the defect-filling material must simultaneously integrate with two distinct tissues: cartilage superficially and bone at the base [44]. Gap-filling adhesive hydrogels positioned between graft edges and host tissues address this challenge by eliminating dead space and bonding to both cartilage and bone. A recent study by Zhou et al. described an immune-modulated adhesive hydrogel specifically designed for osteochondral defects [45]. The system combined HA with catechol-modification (for strong wet adhesion), incorporated dimethyloxalylglycine (DMOG, HIF-1α stabilizer promoting bone formation) and IL-4 (macrophage immunomodulation), and achieved lap shear adhesion strength of 180–220 kPa to both cartilage and bone surfaces. In a rabbit osteochondral defect model, the adhesive hydrogel filled between OATS graft edges and host tissue, resulting in nearly complete bony integration and significantly superior cartilage repair quality compared to non-adhesive control grafts.

The dual-surface adhesion challenge requires materials that bond effectively to cartilage’s highly hydrated, proteoglycan-rich surface and bone’s mineralized, collagen-rich matrix tissues with opposite surface charge characteristics [46]. Few materials achieve strong adhesion to both; most adhesive systems optimize for one tissue at the expense of the other. The most promising approach involves functional diversity within the adhesive material, combining multiple adhesion mechanisms (e.g., catechol-mediated wet adhesion plus NHS ester covalent bonding plus electrostatic interaction) such that different mechanisms dominate at cartilage versus bone interfaces [45].

### 4.4. Adhesive Hydrogels as Standalone Repair Platforms

**Figure 3:**
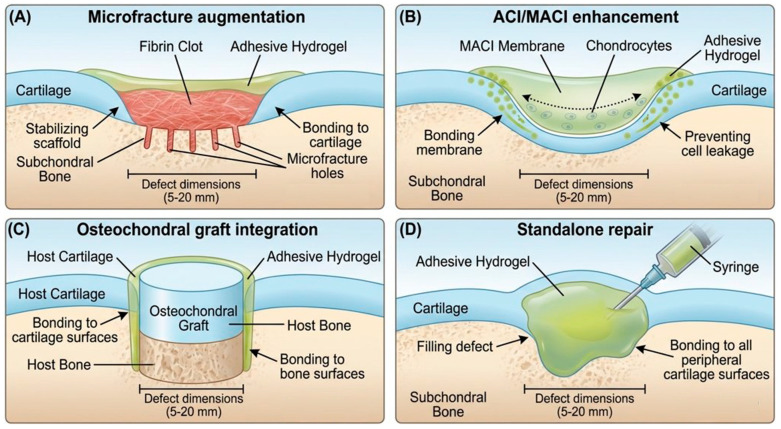
Adhesive hydrogel application across surgical scenarios. (**A**) Microfracture augmentation: clot stabilization and cartilage edge bonding. (**B**) ACI/MACI enhancement: membrane fixation and cell leakage prevention. (**C**) Osteochondral graft integration: simultaneous adhesion to cartilage and bone surfaces. (**D**) Standalone repair: injectable defect-filling with peripheral cartilage bonding.

The ultimate application of adhesive hydrogels is as standalone repair materials that simultaneously serve as fixation scaffold and regeneration matrix, eliminating the need for ancillary devices or surgeries (Figure 3) [31]. This approach represents a paradigm shift from thinking of adhesion as an adjunct property to recognizing it as the central functional requirement of cartilage repair materials. A hypothetical standalone adhesive hydrogel for cartilage repair would directly bond to cartilage defect edges upon injection or application, providing immediate fixation without sutures. Simultaneously, it would provide a three-dimensional environment for chondrocyte or stem cell expansion, growth factor incorporation, and tissue regeneration.

This approach offers multiple clinical advantages. First, it reduces operative complexity, with no need for periosteal flap harvesting or osteochondral graft procurement. Second, it eliminates mechanical fixation complications. Third, it allows standardization; unlike autografts or autologous cell therapies, adhesive hydrogel compositions can be standardized and mass-produced. Fourth, allogeneic or xenogeneic cell sources could be incorporated, broadening accessibility [42].

Implementing standalone adhesive hydrogels requires simultaneous optimization of multiple properties that are often competing: strong wet adhesion, adequate mechanical strength (compressive modulus 0.5–2 MPa to match cartilage), chondrogenic bioactivity, injectable delivery capability, and appropriate degradation kinetics [42]. Few materials have achieved this optimization; most systems excel in some properties while compromising others. Representative adhesive hydrogel systems developed for cartilage repair are summarized in Table 3.

## 5. Material Platforms for Cartilage-Adhesive Hydrogels

### 5.1. Catechol/Dopamine-Functionalized Systems

Mussel-inspired chemistry represents the most celebrated approach in wet adhesion research, inspired by the remarkable adhesive secretions of mussels (Mytilus edulis) that bind permanently to submerged rocks and ship hulls [47]. Mussels achieve this wet adhesion through DOPA, a modified amino acid containing a catechol moiety (two adjacent hydroxyl groups on an aromatic ring). DOPA residues in mussel adhesive proteins undergo oxidation under physiological conditions, generating quinone intermediates that form covalent bonds with tissue nucleophiles and crosslink adjacent protein molecules.

Researchers have synthesized DOPA-modified polymers and catechol-conjugated biomaterials that recapitulate mussel adhesion [47]. Gelatin methacryloyl (GelMA)-DOPA systems—where gelatin is modified with both methacryloyl groups (for photocrosslinking) and DOPA (for adhesion)-achieve excellent adhesion to cartilage, achieving lap shear adhesion strengths of 80–150 kPa [41]. HA-DOPA systems similarly achieve strong adhesion to cartilage due to complementary interactions between HA and cartilage matrix (HA-binding proteins) plus catechol-mediated bonding. These systems offer several advantages: the adhesion mechanism is inspired by nature and validated across diverse tissues, the chemistry is relatively straightforward, and the materials are biocompatible.

However, catechol/dopamine systems face significant limitations. The oxidation of catechol to quinone is uncontrolled; once oxidation begins, it propagates non-specifically throughout the material, generating unwanted browning discoloration [48]. This discoloration is not merely cosmetic—it reflects uncontrolled oxidative polymerization that can generate toxic oxidative byproducts and unstable crosslinks. Moreover, quinone-mediated crosslinks are prone to reduction by biological reducing agents (ascorbic acid, glutathione present in cells and serum), potentially leading to loss of adhesion over weeks to months. Recent studies by Zeng et al. demonstrated that catechol-based adhesives, despite excellent initial adhesion strength (180+ kPa), show substantially reduced adhesion strength after 4 weeks of incubation in cell culture medium containing physiological glutathione concentrations [48].

Enhancement of catechol systems through metal coordination represents an emerging strategy. Iron (Fe^3+^) coordination with catechol generates stable crosslinks, increases adhesion strength, and provides anti-inflammatory activity through iron’s immunomodulatory effects [52]. However, iron coordination introduces biocompatibility concerns; excess iron can induce oxidative stress through Fenton chemistry, and iron leaching from the material raises toxicity concerns.

### 5.2. Nhs Ester and Aldehyde-Based Covalent Bonding

N-hydroxysuccinimide (NHS) ester chemistry represents a complementary approach to catechol systems, utilizing covalent bond formation between activated esters and primary amines (-NH2) abundant in collagen and proteoglycans that comprise cartilage extracellular matrix [50]. NHS esters react rapidly with tissue amines; conjugation occurs in seconds to minutes, depending on amine concentration and pH. This rapid kinetics offers advantages for intraoperative use—the adhesive material can be applied and covalently bonded to tissue within the timeframe of the surgical procedure [51].

Aldehyde-amine Schiff base chemistry provides alternative covalent bonding, particularly relevant for polysaccharide-based hydrogels where aldehyde groups are generated by periodate oxidation of HA, alginate, or dextran. Oxidized HA creates an aldehyde-functionalized polymer that reacts with tissue amines to form reversible imine bonds (Schiff base) initially, which can be further stabilized through reduction or borohydride treatment. Adhesion strength of Oxidized HA -based systems ranges from 40 to 100 kPa, and the mechanism is generally considered more biocompatible than quinone-based systems because aldehyde intermediates are more specific (reacting only with amines) compared to quinones (which undergo non-specific reactions) [53].

A critical trade-off governs NHS ester and aldehyde-based systems: the reactivity that enables rapid bonding carries potential cytotoxicity [53]. Unreacted NHS esters generate free active ester groups that can oxidatively damage nearby cells; aldehyde groups at high concentrations are mutagenic and trigger apoptosis. Balancing adequate reactivity for tissue bonding against cytotoxicity requires careful design—generally keeping aldehyde concentrations below 50 mM and NHS ester below 100 mM [51]. This constraint may limit the achievable adhesion strength compared to less reactive but less toxic systems [49].

### 5.3. Photo-Crosslinkable Adhesive Systems

Photo-initiated crosslinking offers precise temporal and spatial control. Adhesion activates only when illuminated, allowing surgeons to position material before triggering gelation [39,49]. GelMA, a widely used photocrosslinkable platform, can be modified with adhesive peptides or catechol conjugates to provide adhesion while simultaneously enabling photocrosslinking [49]. HAMA (hyaluronic acid methacryloyl) and SilMA (silk fibroin methacryloyl) similarly support both photo-initiated crosslinking and adhesion modification.

The advantage of photocrosslinkable systems is precise control: gelation and adhesion activation occur on demand within seconds, allowing surgical shaping and positioning before the material stiffens. This is particularly relevant for arthroscopic applications where material must be delivered through small portals and shaped within the constrained joint space [54]. The disadvantage is the requirement for light exposure; ultraviolet (UV) light penetrates only 100–300 µm into tissue, limiting the thickness of material that can be cured and raising concerns about phototoxicity. Visible light (wavelength 550–650 nm) penetrates more deeply (500–1000 µm) but requires eosin or other visible-light photosensitizers that may have independent biological effects.

Tyrosine-mediated radical coupling represents a bioorthogonal photochemistry approach, where horseradish peroxidase (HRP) and hydrogen peroxide catalyze the coupling of tyrosine residues through radical formation [55]. This system offers superior biocompatibility (avoiding synthetic photosensitizers) and deeper tissue penetration but requires careful control of reactive oxygen species generation to prevent oxidative damage.

### 5.4. Supramolecular and Physical Adhesion Systems

Supramolecular approaches employ non-covalent interactions—host-guest chemistry, electrostatic interactions and hydrogen bonding-to generate adhesion, offering advantages of reversibility and self-healing while avoiding potential cytotoxicity of covalent chemistries [56]. Host-guest systems based on cyclodextrin (CD) or cucurbit[n]uril (CB[n]) create reversible adhesion through inclusion complex formation; adhesion strength is lower than covalent systems (30–70 kPa typically) but remains sufficient for some applications [57]. The advantage of reversibility is intriguing for revision surgery-theoretically, a reversible adhesive could be debonded without tissue damage, allowing removal and replacement.

Electrostatic adhesion between cationic hydrogels (such as chitosan) and anionic cartilage matrix (rich in proteoglycans and GAGs) provides weak but non-toxic adhesion [58]. This mechanism underlies BST-CarGel’s function and provides adhesion strength of 50–90 kPa [32,33]. Hydrogen bonding networks in polyacrylamide, polyvinyl alcohol (PVA), and related polymers generate adhesion through interfacial hydrogen bond formation. These systems are generally considered biocompatible and non-toxic [56]. However, adhesion strength is modest (20–60 kPa), and stability is compromised by hydration (water molecules compete for hydrogen bonding).

### 5.5. Multi-Mechanism Hybrid Approaches

Recent advances have embraced the concept of “tough adhesion,” advancing beyond single-mechanism adhesion to hybrid systems that combine multiple bonding modes to achieve both high adhesion strength and damage tolerance [29,59]. The seminal work of Yuk et al. demonstrated that combining dissipative interactions at the bulk interface with multiple adhesion mechanisms at the interface itself generates adhesion exceeding 500 kPa in synthetic hydrogels. For cartilage applications, similar logic suggests hybrid systems combining (1) catechol-mediated wet adhesion, (2) covalent NHS ester bonding, (3) electrostatic interaction with proteoglycans, and (4) mechanical interlocking through surface roughness would optimize adhesion strength while improving adhesion durability [29].

The mechanistic basis of the adhesion-versus-biocompatibility trade-off is now analyzed quantitatively. The same nucleophile-reactive chemistries (NHS ester, quinone, aldehyde) that drive the strongest covalent wet adhesion also generate reactive oxygen species or deplete lysine residues on chondrocyte membrane proteins. Across four representative platforms, the resulting trade-off is as follows. (i) Electrostatic chitosan-GP systems (BST-CarGel) achieve 50–90 kPa with chondrocyte viability > 90% but fall below the load-bearing threshold. (ii) Catechol/DOPA-GelMA systems achieve 80–150 kPa with viability 75–90%, with ROS-mediated cytotoxicity from uncontrolled quinone oxidation. (iii) NHS ester/aldehyde covalent systems achieve 60–140 kPa (NHS) or 40–100 kPa (aldehyde) with viability 50–80%; the strongest covalent durability is paid for in chondrocyte survival. (iv) Multi-mechanism hybrid systems combining catechol + Fe^3+^ coordination + supramolecular host–guest interactions are currently the only platforms achieving strength ≥ 100 kPa (150–300 kPa range) with viability ≥85%, by distributing reactivity across orthogonal modes. ROS scavenging, post-application quenchers (free lysine, serum amines), and orthogonal-mode hybridization are the three most evidence-supported mitigation strategies. Detailed cross-platform comparison appears in Table 4 of the response document.

**Figure 4:**
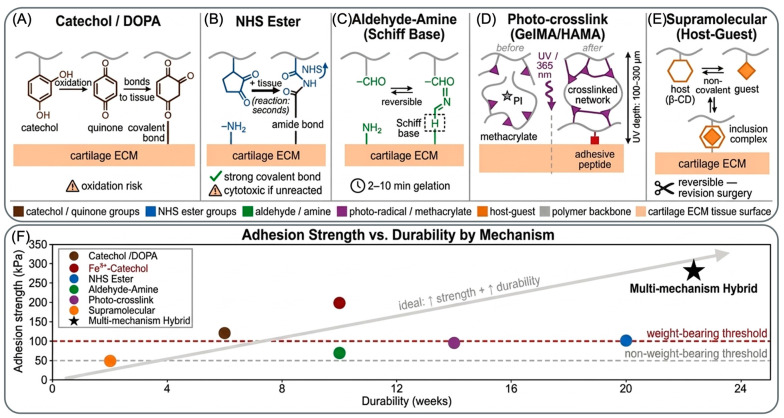
Chemical adhesion mechanisms at the hydrogel–cartilage interface. (**A**) Catechol/DOPA oxidation. (**B**) NHS ester–amine covalent bonding. (**C**) Aldehyde–amine Schiff base formation. (**D**) Photo-crosslinking (GelMA/HAMA). (**E**) Supramolecular host–guest complexation. (**F**) Adhesion strength versus durability by mechanism; multi-mechanism hybrid approaches exceed clinical thresholds over the longest timescale.

Protein-engineered adhesives represent another hybrid approach, utilizing recombinant protein expression to encode multiple functional domains within a single protein scaffold [60]. A hypothetical cartilage adhesive protein might contain: an N-terminal adhesion domain (e.g., mussel foot protein-derived sequences), a middle structural domain providing mechanical strength, and a C-terminal chondrogenic signaling domain (e.g., TGF-β or BMP binding motifs). Such multi-functional proteins, while requiring sophisticated synthesis and quality control, offer unparalleled integration of adhesion, mechanics, and bioactivity (Figure 4) [60]. A comprehensive comparison of these adhesive material platforms is provided in Table 5.

## 6. Injectable Adhesive Hydrogels—Toward One-Step Cartilage Repair

### 6.1. Design Criteria for Injectable Adhesive Systems

Injectable delivery represents the clinical gold standard for cartilage repair, enabling minimally invasive arthroscopic administration and defect conformability without a second surgical site [31,61,62]. Injectable adhesive hydrogels must meet stringent design criteria: they must flow through needles (18–25 gauge for arthroscopic delivery) without segregation or phase separation, gelate within the surgical timeframe (30 s to 5 min), and achieve adequate adhesion strength following gelation. These requirements are often contradictory; materials that flow easily (low viscosity) typically gel slowly, while materials that gel rapidly are difficult to inject.

The shear-thinning property, where apparent viscosity decreases under mechanical shear stress (as occurs during injection), is essential for injectable systems. Shear-thinning enables low viscosity during needle injection (minimizing injection force) while maintaining gel-like consistency upon standing. This property is achievable through several mechanisms: electrostatic interactions in polyelectrolyte systems, where shear disrupts ionic bonds temporarily but they reform upon stress removal; hydrogen bonding networks in polymers like PVA that partially disrupt under shear; or physical interactions in systems with supramolecular assembly [61,63].

Self-healing capacity, where the material repairs localized damage or tearing, is advantageous for injectable systems injected into confined joint spaces where defects may undergo compressive damage before full gelation. Materials that self-heal can recover strength after minor mechanical trauma, maintaining structural integrity [63]. Self-healing mechanisms include reversible covalent bonds (dynamic covalent chemistry using thioesters or disulfides), supramolecular interactions (hydrogen bonding, metal coordination), or microstructural healing (compartmentalized healing agents) [64].

Post-gelation adhesion development represents an often-overlooked design criterion [65]. Ideally, injectable adhesive hydrogels display rapid bulk gelation (enabling handling and mechanical support) followed by continued adhesion maturation over hours to days (enabling strong final adhesion) [65]. This two-phase kinetics requires dual-crosslinking mechanisms: a rapid mechanism establishing gel structure (photocrosslinking, pH-triggered gelation, or ionic crosslinking) followed by a slower, stronger adhesive bonding mechanism (covalent bond formation via NHS esters or catechol oxidation). Few injectable systems optimize this dual-kinetics design; most prioritize rapid gelation while neglecting post-gelation adhesion maturation.

### 6.2. In Situ Crosslinking Mechanisms Compatible with Adhesion

Enzymatic crosslinking represents a particularly elegant in situ mechanism, employing enzymes naturally present in tissue or added as a second component [55]. HRP catalyzes the coupling of phenolic or aniline-modified polymers in the presence of hydrogen peroxide, generating crosslinks within seconds to minutes, depending on H_2_O_2_ concentration [55]. The HRP/H_2_O_2_ system is biocompatible and has been used clinically in other applications. Transglutaminase (TG), a calcium-dependent enzyme naturally involved in wound healing, catalyzes crosslink formation between glutamine and lysine residues in proteins and modified polymers [66]. TG has the advantage of being activated by physiological calcium concentrations, enabling in situ gelation without exogenous reactants.

Thermo-responsive gelation exploits the temperature-dependent phase transition of polymers like poly(N-isopropylacrylamide) (PNIPAM) or chitosan-glycerol phosphate that undergo rapid sol–gel transition at physiological temperature [67]. These polymers are soluble at room temperature but aggregate above their lower critical solution temperature (LCST, typically 32–38 °C), forming hydrogels upon injection into the 37 °C joint. The advantage is simplicity; no additional reagents are required beyond the polymer solution itself. The disadvantage is that gelation kinetics are difficult to control precisely; minor variations in polymer concentration or composition can dramatically alter LCST and gelation rate [67].

Dual-crosslinking approaches combine rapid physical gelation with slower chemical crosslinking, exemplified by systems combining initial thermo-responsive gelation (establishing bulk structure) with subsequent photochemical or enzymatic crosslinking (strengthening structure and enabling adhesion) [68]. The challenge is engineering the system such that initial rapid gelation does not impede subsequent chemical reaction kinetics.

### 6.3. Combining Adhesion with Bioactive Functionality

Beyond adhesion and mechanical support, injectable adhesive hydrogels can be designed to deliver bioactive molecules promoting chondrogenic differentiation or immunomodulation [69,70]. Growth factor incorporation—where TGF-β, BMP-2, or other growth factors are mixed into the injectable formulation or covalently incorporated—provides soluble bioactivity. The challenge is retention: hydrogels are porous, and soluble growth factors rapidly diffuse into surrounding synovial fluid, reducing local concentration and bioavailability [69]. Covalent incorporation through peptide conjugation or heparin-mediated sequestration improves retention.

Immunomodulatory adhesive hydrogels represent an emerging frontier, incorporating molecules like DMOG (HIF-1α stabilizer promoting bone formation and angiogenesis) or IL-4 (macrophage immunomodulation toward M2 reparative phenotype) directly into the adhesive matrix [45]. Zhou et al. demonstrated that HA-catechol adhesive gels incorporating DMOG and IL-4 achieved superior osteochondral defect healing compared to adhesive gels lacking these bioactive molecules, suggesting synergism between mechanical adhesion and immunomodulation.

**Figure 5:**
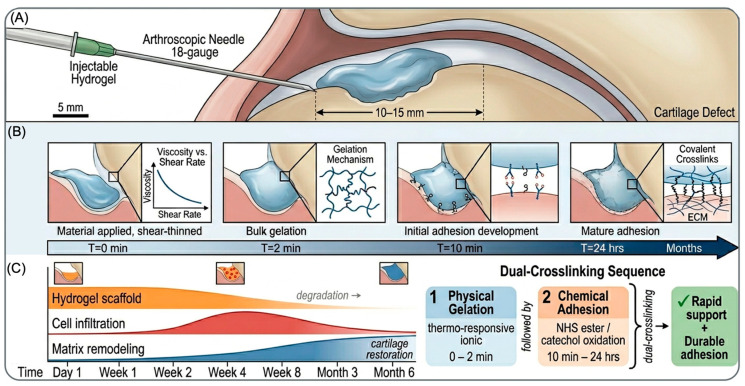
Injectable adhesive hydrogel delivery, gelation, and tissue integration. (**A**) Step 1. Arthroscopic injectable delivery: arthroscopic administration via 18-gauge needle. (**B**) Step 2. Gelation and adhesion development: sequential gelation progression from shear-thinned liquid (T = 0 min) through bulk gelation (T = 2 min), initial adhesion (T = 10 min), and mature bonding (T = 24 h) via dual-crosslinking. (**C**) Step 3. long-term tissue integration: concurrent scaffold degradation, cell infiltration, and matrix remodeling toward cartilage restoration.

Cell delivery within injectable adhesive hydrogels offers the possibility of one-step cell therapy: injecting chondrocytes or MSCs suspended in adhesive hydrogel, with gelation occurring in situ while cells reside within the three-dimensional matrix [71]. This approach combines the benefits of cell therapy (cell source providing chondrogenic factors and matrix synthesis) with mechanical adhesion benefits [31,71]. The critical challenge is biocompatibility of gelation conditions with cell viability; rapid chemical crosslinking (NHS esters, photopolymerization) often compromises cell survival, while gentler biopolymer systems may lack adhesion strength (Figure 5) [32,69].

## 7. Translational Barriers and Future Perspectives

### 7.1. Key Translational Challenges

The progression from promising laboratory findings to clinical translation of adhesive hydrogels faces substantial barriers, many of which are specific to the cartilage repair context [31]. The most critical barrier is a profound knowledge gap regarding long-term adhesion durability under physiological loading. The vast majority of adhesive hydrogel studies, including those developing systems specifically for cartilage repair, assess adhesion strength at a single time point, immediately after gel maturation, typically at 24 h post-gelation. Only a minority of studies examine adhesion strength beyond one week, and essentially no studies comprehensively document adhesion durability beyond three months (though cartilage repair timelines extend 12–24 months) [31]. The few studies examining long-term adhesion durability reveal concerning trends: adhesion strength declines progressively with time, often losing 50% of initial strength by 8–12 weeks [28].

Fatigue testing, the single most important predictor of clinical adhesive durability, remains absent from most developmental adhesive hydrogel studies. While standard ASTM protocols exist for adhesive fatigue testing, their application to hydrogel adhesives has been limited [15]. Available fatigue data are alarming: adhesive hydrogels that achieve impressive acute adhesion strengths (100–150 kPa) often retain only 20–40 kPa after 100,000 load cycles (equivalent to ~2 weeks of physiological loading) [15]. This suggests that acute adhesion strength, which dominates current research literature, provides a severely misleading estimate of clinical adhesion durability. The fatigue and enzymatic stability of representative adhesive systems are summarized in Table 6.

Of the published cartilage-adhesive platforms reviewed in Section 4 and Section 5, only BST-CarGel has multi-year (≥5-year) clinical durability data [32,34]; cyclic-loading benchmarks adapted from ASTM F2255/F2256 are essentially absent for cartilage-adhesive hydrogels; and most in vitro enzymatic challenges use simple buffers rather than synovial fluid or MMP-spiked media. We therefore propose a minimum durability dataset for future cartilage-adhesive studies: (i) cyclic shear/peel testing to ≥1000 cycles at physiologically relevant amplitude (≥0.5–1.0 MPa peak interfacial shear); (ii) accelerated synovial-fluid soak with MMP-2/MMP-9 quantification of degradation kinetics; and (iii) at least one large-animal load-bearing in vivo time point at ≥6 months. Until these data are available, claims of clinically adequate adhesion durability remain extrapolations from acute test geometries to a 24-month physiological loading environment.

The trade-off between adhesion strength and biocompatibility represents another fundamental barrier [28]. The most effective adhesion mechanisms—robust covalent crosslinking with activated esters or aldehydes—are inherently more cytotoxic than biocompatible alternatives. Achieving adequate adhesion strength while maintaining cell viability within the healing defect requires a careful balance that few materials have achieved. Cell-laden adhesive hydrogels face particularly acute versions of this trade-off; incorporating living cells within an actively gelling material requires gelation conditions so mild that adhesion strength often becomes clinically inadequate [71].

Batch-to-batch variability in natural polymer-based adhesives significantly complicates translation [28]. Chitosan, for example, exhibits variable deacetylation degree depending on source and processing conditions, leading to highly variable adhesion strength and gelation kinetics. HA from different suppliers or different extraction batches can vary in molecular weight, degree of modification, and consequently in gel properties [72]. While synthetic polymer adhesives (PEG-based systems) show superior batch consistency, they often suffer from lower biocompatibility or adhesion strength compared to natural polymers [28].

Animal model limitations represent a significant translational barrier that is frequently underappreciated [73]. Most adhesive hydrogel studies employ small animal models (rodents, rabbits) where cartilage defects are small (typically 3–4 mm diameter, corresponding to 10–15 mm^2^ surface area). Clinically relevant cartilage defects are substantially larger, 10–30 mm in diameter (75–700 mm^2^ surface area), imposing different stress distributions and mechanical demands. Additionally, small animal knees lack the congruity and load-bearing demands of human knees; mechanical loading patterns are qualitatively different [74]. Large animal models (sheep, goats, horses) are more clinically relevant but are substantially more expensive and technically challenging, explaining their relative rarity in the adhesive hydrogel literature.

Sterilization compatibility represents a practical but critical barrier often overlooked in early-stage research [75]. Adhesive hydrogels containing reactive functional groups (NHS esters, aldehydes, catechols) are susceptible to degradation during gamma irradiation sterilization, the industry standard for medical devices. Gamma irradiation generates free radicals that can oxidize catechols, cleave Schiff bases, and degrade the polymer backbone, resulting in dramatic loss of adhesion strength and altered gelation kinetics. While alternatives such as ethylene oxide (EtO) sterilization exist, they are less frequently available and raise toxicity concerns if residual EtO remains. Some adhesive systems require validation of post-sterilization adhesion strength; most lack this critical validation [75].

The shelf-life of adhesive hydrogels containing reactive functional groups is substantially limited compared to inert polymer scaffolds. Catechol-conjugated polymers oxidize in air and solution, losing adhesive functionality within weeks to months [75]. NHS ester-modified polymers hydrolyze slowly in aqueous solution, with an activity half-life of weeks [50]. Achieving multi-year shelf-life, the standard expectation for clinical devices, requires sophisticated stabilization strategies (inert gas packaging, desiccation, pH buffering) that add complexity and cost.

### 7.2. Regulatory Considerations

The regulatory classification of adhesive hydrogels for cartilage repair remains ambiguous, creating uncertainty for manufacturers regarding the regulatory pathway and required evidence [42]. An adhesive hydrogel for cartilage repair could reasonably be classified as: (1) a scaffold device, (2) a drug-device combination product (requiring clinical efficacy evidence), or (3) an ATMP if cell-laden, requiring complex regulatory review in Europe. This classification ambiguity has prevented clinical development of otherwise promising adhesive systems [42].

Cell-laden adhesive hydrogels face a particularly complex regulatory status [42]. In the European Union, these products qualify as ATMPs, requiring Committee for Advanced Therapies (CAT) review and accelerated drug development pathways. In the United States, similar products fall under the FDA’s Regenerative Medicine Advanced Therapy (RMAT) designation, providing expedited review pathways but still requiring substantial clinical evidence. The regulatory burden of ATMP classification has substantially slowed clinical translation of cell-based adhesive systems.

Acellular adhesive hydrogels may achieve simpler regulatory approval, potentially through 510(k) pathways if they can be positioned with appropriate predicate devices [76]. However, identifying relevant predicates is challenging; current regulatory-approved cartilage repair products (BST-CarGel, MACI (Vericel Corporation)) established relatively low regulatory bars, and new products claiming improved efficacy will face higher scrutiny. The FDA generally requires clinical efficacy evidence for adhesive systems marketed as improving on existing approaches [76].

In practice, 510(k) clearance is unrealistic for novel cartilage-adhesive hydrogels because no substantially equivalent predicate exists; both BST-CarGel^®^ (Smith & Nephew/Anika Therapeutics; CE-marked Class III since 2009; FDA pathway pursued via HDE/PMA) and GelrinC^®^ (CE-marked) are themselves Class III scaffold devices that established the precedent. The realistic FDA routes for acellular adhesive scaffolds are therefore De Novo or PMA, accompanied by ISO 10993 biocompatibility, GLP large-animal, and clinical evidence. Cell-laden adhesive constructs follow the BLA pathway under FDA’s CBER + 21 CFR 1271 framework (with RMAT designation as a possible accelerator), with MACI^®^ (Vericel; FDA BLA 2016) [77], Spherox^®^ (CO.DON; EMA centralized approval 2017 [78] as Tissue-Engineered Product ATMP), and the ChondroCelect withdrawal (2014; reimbursement-and-manufacturing precedent) [77] as the principal trajectories. Growth-factor-loaded adhesive hydrogels follow the combination-product pathway, with Augment^®^ Bone Graft (rhPDGF-BB; FDA PMA 2015) [79] the closest analogous precedent. No existing approved product covers patient-specific bioprinted cell-laden adhesive constructs. These regulatory pathways for acellular and cell-laden adhesive hydrogels are summarized in Table 7.

### 7.3. Future Directions

Overcoming these translational barriers will require not only incremental material improvements but also paradigm-level innovations across fabrication, computational design, and surgical integration. Four-dimensional (4D) bioprinting represents an emerging opportunity to engineer adhesive hydrogels with spatiotemporal control of material properties [80]. Current 3D bioprinting uses computer-controlled deposition of hydrogels to generate complex geometries; 4D bioprinting adds a temporal dimension by engineering material properties to change over time. Applied to cartilage adhesion, 4D bioprinting could generate adhesive constructs where adhesion is strongest at peripheral interfaces (bonding to native cartilage) while interior regions remain more permeable and degradable (promoting neovascularization and infiltration) [81].

Artificial intelligence and machine learning present untapped opportunities for adhesive formulation optimization [82]. Adhesive hydrogels are complex multicomponent systems where each component (polymer type, degree of modification, crosslinking density, adhesive group density, growth factor type and concentration) influences final properties [28,82]. The interaction space is enormous; traditional design-of-experiments approaches are impractical for comprehensive optimization. Machine learning models trained on large datasets of adhesive hydrogel properties could predict optimal formulations for specific clinical applications, dramatically accelerating development.

To distinguish demonstrated evidence from extrapolation, we categorize the proposed future tools as follows. (a) Demonstrated specifically in cartilage adhesive systems: only Sharma et al. from photoreactive adhesive-hydrogel composite tested in human cartilage repair and Zhou et al. from immune-modulated catechol osteochondral adhesive with rabbit in vivo data constitute rationally designed cartilage-specific adhesive systems with cartilage in vivo data [39,45]. To our knowledge, no published study has applied modern machine-learning formulation optimization (Bayesian optimization, generative models) to a cartilage adhesive specifically, and our advocacy of these tools is on rationale-by-extrapolation grounds. (b) Demonstrated in adjacent musculoskeletal-adhesive contexts and concretely transferable: Freedman et al.’s tough adhesive for tendon repair is the closest demonstrated musculoskeletal-adhesive analog; AI-based hydrogel design surveyed by Li et al. does not address cartilage adhesion directly [82,83]. (c) Currently, conceptual for cartilage adhesives: 4D shape-morphing adhesives from Kirillova et al. and patient-specific bioprinted constructs from Daly et al. remain conceptual at the cartilage-adhesive interface, with manufacturing, GMP, sterilization, and reimbursement barriers that must be resolved before clinical translation [80,81]. We therefore advocate these tools as research directions whose feasibility has been established in adjacent domains, not as imminent clinical platforms.

Smart adhesion—engineered systems enabling controlled bonding and debonding on demand—represents a moonshot goal with significant clinical implications [83]. If adhesive bonds could be reversibly activated and deactivated, revision surgery would become feasible without traumatic tissue damage. Current cartilage repair revisions often fail because removing failed repair tissue damages the surrounding native cartilage [83]. Reversible adhesive systems using trigger mechanisms (light-responsive, pH-responsive, enzyme-responsive) could allow complete removal and replacement without iatrogenic injury.

Patient-specific adhesive hydrogels matched to individual defect geometry and joint biomechanics represent the ultimate personalized medicine approach [81,82,84]. 3D joint imaging could define defect geometry with submillimeter precision, while gait analysis could quantify individual loading patterns. Computational modeling could then predict optimal adhesive formulation for that specific patient. Personalized hydrogels could be manufactured in-house at regional hospitals, delivered to surgeons pre-customized for each patient [81,82]. Realizing this vision will require advances in point-of-care manufacturing, regulatory frameworks for personalized products, and standardized outcome metrics.

### 7.4. Conclusions

Articular cartilage repair has long been constrained by a fundamental and underappreciated problem: the absence of active biological adhesion between repair materials and host tissue. This review has demonstrated that the clinical failures of microfracture, ACI/MACI, and osteochondral grafting, despite their mechanistic diversity, converge on a common denominator of inadequate scaffold-tissue integration. Adhesive hydrogels directly address this unmet need by transforming passive space-filling scaffolds into active bonding platforms capable of resisting the demanding biomechanical environment of the articular joint.

The material landscape surveyed here, spanning mussel-inspired catechol systems, NHS ester covalent bonding, photocrosslinkable platforms, supramolecular approaches, and multi-mechanism hybrid designs, collectively demonstrates that wet adhesion strengths sufficient for clinical application (>100 kPa for weight-bearing sites) are achievable. Clinically validated systems such as BST-CarGel have already confirmed that augmenting biological adhesion translates to measurable improvements in repair tissue quality at the population level. Injectable formulations designed for arthroscopic delivery further reduce the barrier to clinical adoption by enabling one-step, minimally invasive repair without secondary surgical sites.

Nevertheless, a substantial translational gap remains. Long-term adhesion durability under cyclic physiological loading, biocompatibility-adhesion trade-offs in cell-laden systems, sterilization compatibility, batch-to-batch consistency, and regulatory classification ambiguity collectively constitute barriers that laboratory benchmarks have not yet resolved. The field must move beyond acute adhesion strength as its primary metric and embrace fatigue testing, degradation-coupled adhesion measurements, and synovial fluid-conditioned testing as standard practice.

**Figure 6:**
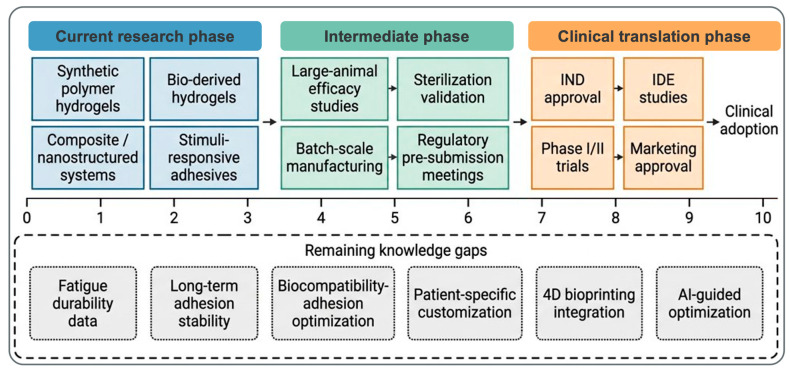
Translational roadmap from preclinical research to clinical implementation.

Looking ahead, the convergence of 4D bioprinting, AI-guided formulation design, and smart reversible adhesion systems offers a compelling roadmap for next-generation cartilage repair platforms. Patient-specific adhesive constructs—matched to individual defect geometry, loading environment, and biological profile—represent the aspirational endpoint of this trajectory. Achieving this vision will require coordinated effort across materials science, tissue engineering, regulatory science, and orthopedic surgery. Adhesive hydrogels are not a supplementary adjunct to cartilage repair; they are the missing link that the field has long required, and their clinical integration represents the most promising near-term pathway toward durable, biologically active joint restoration (Figure 6).

## Figures and Tables

**Table 1:** Comparison of Current Cartilage Repair Techniques: Procedure, Mechanism, and Integration Challenges.

Procedure	Mechanism	Primary Adhesion Failure	Failure Rate at 5 Years (%)	Citation
Microfracture	Subchondral bone penetration, fibrin clot formation	Fibrin clot washout by synovial fluid and mechanical shear; incomplete cartilage-cartilage integration	50–75 (fibrocartilage)	[7,8]
ACI/MACI	Autologous chondrocyte expansion and implantation; periosteal/collagen membrane coverage	Periosteal/membrane delamination; cell leakage; incomplete lateral integration with native cartilage	15–30 (functional failure)	[9,14]
OATS	Osteochondral autograft plug transfer; structural fill	Gap formation at graft-host interface; dead space; micromotion; incomplete adhesion	20–40 (subsidence)	[12,14]
Allograft (fresh)	Fresh cadaveric osteochondral graft; structural fill	Similar to OATS; gap formation; additional immunologic concerns	25–45 (integration failure)	[13,14]

**Table 2:** Adhesion Test Methods and Clinical Relevance for Cartilage Repair.

Test Method	Stress Type	Standard Values (kPa)	Clinical Relevance	Citation
Lap shear	Pure shear	50–500 (tissue-dependent)	Directly reflects the joint shear environment. most clinically relevant	[15,27]
Tensile pull-off	Perpendicular tension	30–300 (material-dependent)	Reflects delamination forces; relevant for peripheral integration	[15,28]
Peel test	Progressive interface separation	10–200 (N/m width)	Sensitive to interface heterogeneity; mimics progressive edge failure	[15,28]
Adhesive failure threshold (estimated)	Mixed mode (shear + tension)	>100 (weight-bearing)	Conservative estimate for permanent, load-bearing adhesion	[29]
20–50 (non-weight-bearing)	Sufficient for temporary fixation or non-load-bearing applications			[15]

**Table 3:** Summary of Adhesive Hydrogel Systems for Cartilage Repair Categorized by Surgical Application.

System/Platform	Material Composition	Adhesion Mechanism	Adhesion Strength (kPa)	Surgical Application	Citation
BST-CarGel	Chitosan-GP	Electrostatic (chitosan-GAG)	50–90	Microfracture augmentation	[32,33,34]
GelrinC	PEG-fibrinogen	Covalent (UV-crosslink) + fibrin binding	70–110	Microfracture augmentation	[36,37]
HA-Catechol	HA-DOPA	Catechol-mediated wet adhesion	80–150	ACI/MACI enhancement; standalone repair	[41,47,48]
PHE-Gel	HA-catechol + DMOG/IL-4	Catechol + immunomodulation	180–220	Osteochondral graft integration	[45]
GelMA-peptide	Gelatin methacryloyl + adhesive peptides	Peptide-mediated + photocrosslink	60–130	ACI/MACI; standalone repair	[41,49]
Ox-Alginate-NHS	Oxidized alginate + NHS ester	Covalent (Schiff base + NHS)	40–100	Microfracture augmentation; clot stabilization	[50,51]

**Table 4:** Adhesion Strength versus Chondrocyte Biocompatibility Across Adhesive Material Platforms.

Material Platform	Adhesion Strength (kPa)	Chondrocyte Viability (%)	Cytotoxicity Mode	Citation
BST-CarGel (chitosan-GP)	50–90	>85	None reported; mild leukocyte recruitment	[32,34,58]
Catechol/DOPA (GelMA)	80–150	75–90	Quinone/ROS-mediated membrane damage; H_2_O_2_ generation	[7,48,60]
NHS ester/aldehyde covalent (Shiff)	60–140/40–100	50–80 (concentration-dependent)	Free NHS ester protein crosslinking; aldehyde mutagenicity at high conc.	[50,51,53]
Multi-mechanism hybrid (catechol + Fe^3+^ + supramolecular)	150–300	85–95 (Select systems; preclinical)	Minimal-reactivity diluted across modes	[29,45,52]

**Table 5:** Comprehensive Comparison of Adhesive Material Platforms for Cartilage Applications.

Material Platform	Adhesion Mechanism	Strength (kPa)	Gelation Time	Durability	Citation
Catechol/DOPA	Quinone crosslink + wet adhesion	80–150	5–10 min	Moderate (4–8 weeks); susceptible to reduction	[47,48]
Fe^3+^-Catechol	Metal coordination + wet adhesion	150–250	3–5 min	Good (8–12 weeks); iron biocompatibility concerns	[48,52]
NHS Ester	Covalent amine bonding	60–140	1–5 s (pre-mixed); <30 s (contact)	Excellent (>6 months); toxicity if unreacted	[50,51]
Aldehyde-Amine (Schiff)	Reversible imine + Schiff base	40–100	2–10 min	Moderate (4–8 weeks); reversibility can be an advantage	[50,51]
Photo-crosslink (GelMA/HAMA)	Photopolymerization + peptide/catechol	60–130	30–120 s (UV); 1–5 min (visible)	Good (>3 months); light penetration limited	[39,49]
Supramolecular (Host-Guest)	Reversible inclusion complex	30–70	Instant (pre-associated); minutes (exchange)	Low (2–4 weeks); reversibility allows debonding	[56,57]
Electrostatic (Chitosan-GAG)	Ionic interaction with cartilage proteoglycans	50–90	10–30 min (thermo-responsive)	Moderate (6–12 weeks); pH/salt sensitive	[33,58]
Multi-mechanism Hybrid	Combined: covalent + coordination + electrostatic	150–300	5–15 min (depending on active mechanisms)	Excellent (>6 months); highest durability	[29,45,52]

**Table 6:** Fatigue and Enzymatic Stability of Representative Cartilage-Adhesive Hydrogel Systems.

System/Material	Test Type & Conditions	Cyclic/Enzymatic Stability	Reported Duration	Initial → Retained Strength	Citation
BST-CarGel (chitosan-GP)	In vivo (sheep/human RCT) under joint loading	Maintained over physiological cyclic load; no in vitro fatigue test	12–60 months (clinical)	Stable hyaline-like fill at 5 years	[32,34]
Catechol/DOPA-GelMA	In vitro lap-shear after PBS/synovial-fluid soak	Strength loss under enzymatic challenge (MMP, hyaluronidase)	4–8 weeks	80–150 kPa; retention variable by formulation	[41,47,48]
Tough adhesive (Yuk et al.)	Acute cyclic peel/lap-shear in PBS; non-cartilage models	Fatigue-resistance over >1000 cycles in non-cartilage models	Acute (days); cartilage data lacking	Shear 120 kPa, tensile 130 kPa (skin/heart)	[29]
Fe^3+^-coordinated catechol	Wet adhesion + simulated synovial fluid	Improved enzymatic resistance vs. catechol-only	8–12 weeks	150–250 kPa to ~70% at 8 weeks	[47,52]
PHE-Gel (HA-catechol + DMOG/IL-4)	Rabbit osteochondral defect, cyclic gait loading	Adhesion maintained ≥ 12 weeks under in vivo cyclic load	12 weeks (in vivo)	180–220 kPa, durable interface	[45]

**Table 7:** Regulatory Pathways for Acellular versus Cell-Laden Adhesive Hydrogels: FDA vs. EU Requirements.

Product Class	FDA Pathway	EMA Pathway	Key Precedent	Citation
Acellular hydrogel	De Novo or PMA (Class III); ISO 10993+ GLP animal+ clinical data	Class III under MDR 2017/745; CE via Notified Body	BST-CarGel^®^ (CE mark; Health Canada 2012)	[32,34]
Cell-laden construct	BLA (CBER); RMAT designation eligible; Phase I–III required	ATMP (Tissue-Engineered Product subcategory); centralized EMA assessment via CAT; full clinical dossier	MACI^®^ (FDA BLA 2016); (EMA 2017); JACC^®^ (PMDA 2012)	[42,77,78]
Growth-factor-loaded combination	Combination product; IND + NDA/BLA for the bioactive moiety	Combination/medicinal product; centralized EMA review	Augment^®^ Bone Graft (rhPDGF-BB; FDA PMA 2015)	[79]

## Data Availability

No new data were created or analyzed in this study. Data sharing is not applicable to this article.
